# Supplementary material for: Role and mechanism of NCAPD3 in promoting malignant behaviors in gastric cancer
Source: Front Pharmacol. 2024 Apr 22;15:1341039. doi: 10.3389/fphar.2024.1341039 (PMC11070777; doi:10.3389/fphar.2024.1341039)
Supplement: Supplementary file 11 [file DataSheet2.ZIP › GSEA/immunologic/my_analysis.Gsea.1599462003817/GSE2706_UNSTIM_VS_2H_LPS_DC_UP.html]

Details for gene set GSE2706\_UNSTIM\_VS\_2H\_LPS\_DC\_UP[GSEA]

|  || Dataset | filtered\_dataset.sample\_info.cls#WT\_versus\_NCAPD3\_MUT |
| Phenotype | sample\_info.cls#WT\_versus\_NCAPD3\_MUT |
| Upregulated in class | WT |
| GeneSet | GSE2706\_UNSTIM\_VS\_2H\_LPS\_DC\_UP |
| Enrichment Score (ES) | 0.4472363 |
| Normalized Enrichment Score (NES) | 2.0462058 |
| Nominal p-value | 0.0015974441 |
| FDR q-value | 0.640204 |
| FWER p-Value | 0.986 |
Table: GSEA Results Summary

  

Fig 1: Enrichment plot: GSE2706\_UNSTIM\_VS\_2H\_LPS\_DC\_UP      
 Profile of the Running ES Score & Positions of GeneSet Members on the Rank Ordered List

  

| SYMBOL | TITLE | RANK IN GENE LIST | RANK METRIC SCORE | RUNNING ES | CORE ENRICHMENT || 1 | 7571 | ZNF23 | 25 | 1.018 | 0.0488 | Yes |
| 2 | 222236 | NAPEPLD | 36 | 0.977 | 0.1058 | Yes |
| 3 | 9489 | PGS1 | 84 | 0.820 | 0.1257 | Yes |
| 4 | 55900 | ZNF302 | 145 | 0.740 | 0.1311 | Yes |
| 5 | 80304 | C2orf44 | 152 | 0.731 | 0.1748 | Yes |
| 6 | 10795 | ZNF268 | 168 | 0.710 | 0.2106 | Yes |
| 7 | 54492 | NEURL1B | 177 | 0.697 | 0.2507 | Yes |
| 8 | 339559 | ZFP69 | 188 | 0.686 | 0.2886 | Yes |
| 9 | 55028 | C17orf80 | 222 | 0.659 | 0.3080 | Yes |
| 10 | 146050 | ZSCAN29 | 226 | 0.654 | 0.3488 | Yes |
| 11 | 91574 | C12orf65 | 238 | 0.646 | 0.3833 | Yes |
| 12 | 6426 | SRSF1 | 243 | 0.637 | 0.4223 | Yes |
| 13 | 25973 | PARS2 | 266 | 0.620 | 0.4472 | Yes |
| 14 | 27079 | RPUSD2 | 320 | 0.582 | 0.4472 | Yes |
| 15 | 115509 | ZNF689 | 374 | 0.551 | 0.4452 | No |
| 16 | 60686 | C14orf93 | 435 | 0.513 | 0.4356 | No |
| 17 | 80095 | ZNF606 | 479 | 0.487 | 0.4365 | No |
| 18 | 55039 | TRMT12 | 599 | 0.424 | 0.3785 | No |
| 19 | 54663 | WDR74 | 628 | 0.408 | 0.3851 | No |
| 20 | 643836 | ZFP62 | 639 | 0.405 | 0.4045 | No |
| 21 | 157570 | ESCO2 | 685 | 0.386 | 0.3974 | No |
| 22 | 8772 | FADD | 742 | 0.355 | 0.3803 | No |
| 23 | 57035 | RSRP1 | 1061 | -0.452 | 0.1806 | No |
| 24 | 5565 | PRKAB2 | 1094 | -0.474 | 0.1886 | No |
| 25 | 57649 | PHF12 | 1212 | -0.588 | 0.1429 | No |
Table: GSEA details [plain text format]

  

Fig 2: GSE2706\_UNSTIM\_VS\_2H\_LPS\_DC\_UP      
 Blue-Pink O' Gram in the Space of the Analyzed GeneSet

  

Fig 3: GSE2706\_UNSTIM\_VS\_2H\_LPS\_DC\_UP: Random ES distribution      
 Gene set null distribution of ES for **GSE2706\_UNSTIM\_VS\_2H\_LPS\_DC\_UP**
